# Factors and Mechanisms Influencing Conjugation In Vivo in the Gastrointestinal Tract Environment: A Review

**DOI:** 10.3390/ijms24065919

**Published:** 2023-03-21

**Authors:** Wei Liu, Yanhu Huang, Han Zhang, Ziyi Liu, Quanmin Huan, Xia Xiao, Zhiqiang Wang

**Affiliations:** 1 College of Veterinary Medicine, Yangzhou University, Yangzhou 225012, China; 2 Jiangsu Co-Innovation Center for Prevention and Control of Important Animal Infectious Diseases and Zoonoses, Yangzhou 225012, China; 3 Institute of Comparative Medicine, Yangzhou University, Yangzhou 225012, China

**Keywords:** horizontal gene transfer, conjugation, plasmid, conjugative frequency, in vivo, intestinal microbiota

## Abstract

The emergence and spread of antibiotic resistance genes (ARGs) have imposed a serious threat on global public health. Horizontal gene transfer (HGT) via plasmids is mainly responsible for the spread of ARGs, and conjugation plays an important role in HGT. The conjugation process is very active in vivo and its effect on the spreading of ARGs may be underestimated. In this review, factors affecting conjugation in vivo, especially in the intestinal environment, are summarized. In addition, the potential mechanisms affecting conjugation in vivo are summarized from the perspectives of bacterial colonization and the conjugation process.

## 1. Introduction

With the emergence and enrichment of antibiotic resistance genes (ARGs), antimicrobial resistance (AMR) poses a major threat to human health worldwide. ARGs such as *mcr-*1, *bla*_NDM_ and *tet*(X) mediating resistance to the last-line antibacterial agents, namely, colistin, carbapenems and tigecycline, respectively [1,2,3,4], severely weaken the effectiveness of antibiotics. In 2019, 4.95 million deaths were associated with bacterial AMR, including 1.27 million deaths that were directly attributable to bacterial AMR [5]. Horizontal gene transfer (HGT) is mainly responsible for the spread of ARGs in the environment [6]. The main methods of HGT are transformation (recipient bacteria capture the DNA exposed outside the host bacteria), transduction (bacteriophage-mediated DNA transfer between bacteria), conjugation (plasmids are transferred from one bacterial cell to another via sexual pili) [7], vesiduction (DNA transfer via extracellular vesicles [EVs]) [8] and transposition (transposons ‘jump’ from one position of the genome to another through a series of processes such as cutting and reintegration) [9].

**Figure 1:**
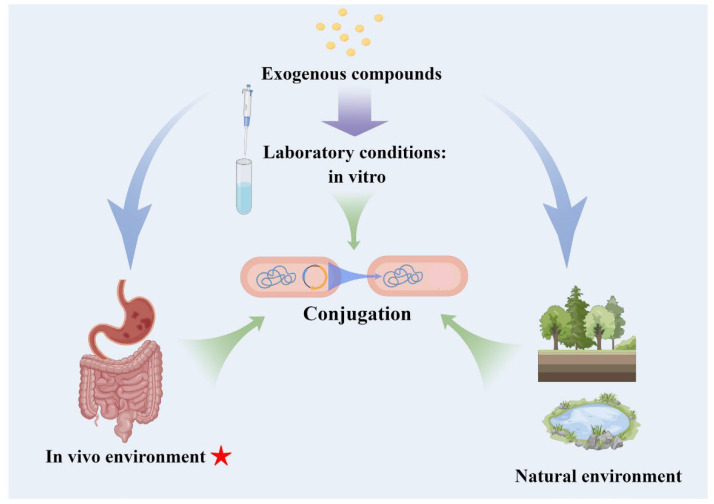
Factors influencing conjugation. There are many factors that promote or inhibit conjugation, not only in laboratory conditions, but also in the natural environment and in vivo environment. Further consideration is needed as to whether some compounds that affect conjugation under laboratory conditions have different effects when they occur in the natural environment or in vivo.

Plasmids are important vectors of ARGs and are involved in the conjugation process. Conjugation is a natural phenomenon among bacterial populations, with a very low conjugative frequency (CF) [10]. However, numerous studies have proved that bacterial conjugative transfer can be promoted or inhibited not only in vitro (laboratory condition, natural environment), but also in vivo (mainly the gastrointestinal tract and so on) (Figure 1) by several factors via different mechanisms, such as the disruption of metabolism, destruction of the cell structure or regulation of related gene expression in the donor and/or recipient cells [11,12,13,14,15]. Understanding the effects of different factors on conjugation may help in the development of more effective strategies to combat the spread of antibiotic resistance.

Factors affecting the conjugation of plasmids have been extensively studied in vitro. These factors include exogenous compounds such as antibiotics used at sub-inhibitory concentrations, non-antibiotic drugs, metal ions and nanomaterials, disinfectants, preservatives and other additives [16,17,18,19,20,21]. In addition, biofilm formation has been reported to contribute to conjugation [22,23]. In previous reviews, exogenous compounds with promoting or inhibitory effects on HGT in vitro have been well summarized, and related mechanisms have been discussed [24]. Another recent review summarized the methods for the determination of HGT in the water environment and the related factors affecting HGT in the natural environment [25]. The effects and mechanisms of emerging pollutants in the environment on the spread of ARGs have also been well reviewed [26]. 

Numerous microorganisms inhabit the oral cavity, upper respiratory tract, gastrointestinal tract and urinary tract of poultry and mammals. In particular, the gastrointestinal tract has a very dense microbial community [27,28,29]. Intestinal microbiota are mainly *Firmicutes* and *Bacteroidetes*, and they also contain opportunistic pathogens, including *Escherichia coli*, *Klebsiella pneumoniae*, *Enterococcus faecalis*, *Enterococcus faecium* and others [30,31]. Thus, the HGT of ARGs is extensively active in vivo. Bacteria may easily acquire ARGs from intra or extra bacteria members of the gut microbiota via the plasmid conjugation process, risking the spread of antibiotic resistance. Hence, it is necessary to identify the factors and mechanisms affecting conjugation in vivo and develop inhibitory strategies based on these factors to prevent the spread of ARGs. In fact, there have been numerous reports about the effects of substances on in vivo conjugation [32,33,34]. Logan C Ott et al. summarized the research model of conjugative transfer from computer simulation in vitro and in vivo, which reflected the real situation in the intestine and laid a foundation for further research on the factors and mechanisms affecting intestinal conjugation [35]. However, few reviews summarize the factors affecting HGT in vivo and its mechanisms. Therefore, factors and mechanisms affecting the spread of ARGs in the gastrointestinal environment also need to be considered.

In this review, we summarize the latest advances in various factors affecting conjugation in vivo, especially in the gastrointestinal tract, and discuss the mechanisms underlying changes in CF. Overall, this review may help to develop novel insights for combating conjugation-mediated HGT in vivo.

## 2. Factors Influencing Conjugation In Vivo

### 2.1. The Density of Donor and Recipient Bacteria

**Figure 2:**
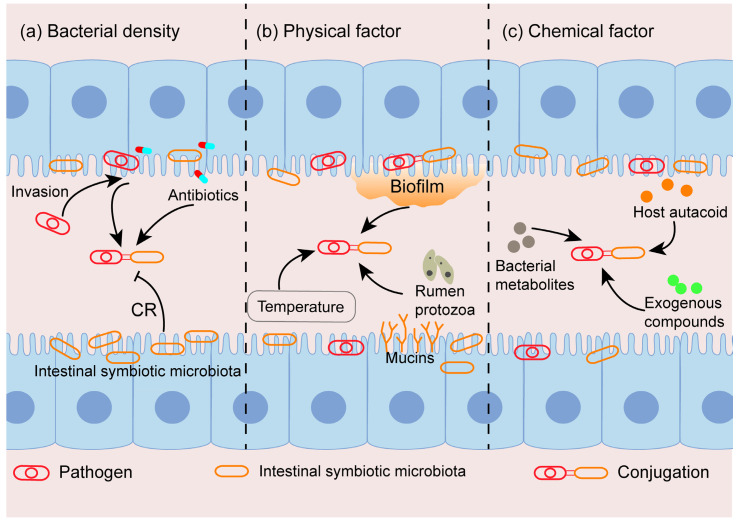
Factors influencing conjugation in vivo. (**a**) Conjugation relies on cell density, as it requires the direct contact of donor and recipient bacteria through the sex pilus to establish a connection bridge. Colonization resistance (CR) mediated by intestinal symbiotic microbiota maintains the density of pathogenic bacteria (donor cells) at a low level, thereby inhibiting conjugation transfer; the invasion of pathogenic bacteria and the selection of antibiotics increase the colonization density of donor and recipient bacteria, which is conducive to conjugation. (**b**) Physical factors affecting conjugation in vivo, including temperature, biofilm formation, mucins, rumen protozoa and anaerobic conditions. (**c**) Chemical factors affecting conjugative transfer in vivo, including exogenous compounds, bacterial metabolites and host autacoid.

In vivo, the gastrointestinal tract contains the highest number and diversity of microorganisms. Studies have clearly shown that the density of donor and recipient bacteria is a key factor affecting the CF in vivo (Figure 2) [36]. *Enterobacteriaceae* are usually a minority in the normal gut, but plasmid conjugation between members of *Enterobacteriales* is particularly frequent. Thus, factors that influence the population of *Enterobacteriales* would greatly influence the CF in the intestinal tract. For example, when *Enterobacteriales* begin to proliferate in large numbers under the influence of specific factors and reach a higher population density, the CF will greatly increase [37]. 

The use of antibiotics not only directly accelerates the development of drug resistance through pressure selection, but also affects the composition of the intestinal microbiota. Studies have shown that antibiotics can affect the composition of the intestinal microbiota in rats and inhibit the *Firmicutes*, resulting in an increase in the population of Proteobacteria, mainly *Enterobacteriales* [38]. Under the treatment of antibiotics, the donor bacteria (cells carrying drug-resistant plasmids) and *Enterobacteriaceae* (the main recipient cell) in the gastrointestinal tract grow in large numbers, thereby promoting plasmid conjugation. For example, the CF of plasmids carrying the *bla*_CMY-2_ gene was significantly increased after the intestinal microbiota was exposed to ampicillin, and the gene maintained a relatively high abundance in the intestine for a long time (Table 1) [39]. Therefore, the selective action of antibiotics against bacteria carrying resistant plasmids and *Enterobacteriales* is a key factor affecting the density of donor and recipient bacteria and the transmission efficiency of ARGs in the gastrointestinal tract.

In addition, some antibiotics can directly promote the CF between bacteria by accelerating the conjugation process [40]. A sub-inhibitory concentration of enrofloxacin can promote the conjugation of IncY plasmid carrying the *qnrS* gene in the chicken intestinal environment (Table 1) [34]. Our previous study found that colistin at 1/8 MIC increased the CF of the RP4-7 plasmid by 38-fold and significantly promoted the conjugative transfer of the IncX3 plasmid encoding *bla*_NDM-5_ and the IncI2 plasmid encoding *mcr-1* to *E. coli* J53 [41]. However, the effect of colistin on conjugative transfer in vivo remains to be further explored. 

In addition to antibiotics, there are still many factors that can affect the structure of the gastrointestinal flora and regulate the density of donor and recipient bacteria, such as diet and disease status. Some diseases, such as inflammatory bowel disease (IBD) and type 2 diabetes, can lead to intestinal microecological imbalance; thus, they may affect the transfer and distribution of ARGs in the intestine [42]. Improving the gastrointestinal environment and optimizing the structure of intestinal symbiotic microbiota are effective methods to prevent the spread of antibiotic resistance in vivo. However, how do the diseases influence the intestinal flora and then affect the CF? What kinds of bacteria are involved in the plasmid conjugation process? Which are the donor and recipient bacteria? Which bacteria is key for the plasmid conjugation process in vivo? All these issues are key for HGT in vivo and need further exploration.

### 2.2. Physical Factor

The intestine is a complex environment, and the physical environment is relatively stable. The disequilibrate of the physical environment would affect the HGT in the bacterial community, including the changes of temperature, biofilm formation and others in the gastrointestinal environment (Figure 2 and Table 1). 

#### 2.2.1. Temperature

An increasing number of studies have shown that temperature is one of the key factors affecting conjugative transfer. Most studies on plasmid conjugative transfer focus on 37 °C, the optimal survival temperature of bacteria, which is also similar to the body temperature of most mammals. Studies have shown that in chicken meat, the CF at 37 °C is higher than that at 25 °C [43]. Generally, the closer the temperature is to 37 °C, the higher the CF. In previous studies, different temperatures have different effects on the CF [44,45]. However, most studies have focused on the natural environment, where changes in gastrointestinal temperature are much smaller than in the natural environment. For example, the body temperature of mammals cannot drop below 25°C for normal survival. Therefore, it remains to be explored whether subtle changes in gastrointestinal temperature affect the conjugation of mammals in abnormal conditions, such as heatstroke and weakness.

#### 2.2.2. Biofilm Formation

Biofilms are bacterial communities that adhere to living or non-living surfaces and may also exist as free-floating communities [46]. Bacteria tend to form biofilms on organs or medical devices such as the teeth, heart, lungs and catheters. A classic example is bacterial endocarditis, which is primarily caused by *Streptococcus* and *Staphylococcus* spp. Inflammatory damage stimulates the formation of insoluble fibrin and platelet clots, which facilitate bacterial colonization [46]. The formation of biofilms provides a stable platform for resident pathogens, thus increasing resistance to antibiotic treatment and host immune responses [47]. Studies have shown that the CF is higher in biofilms. On the one hand, the density of bacteria in the biofilm is higher, and the chance of cell-to-cell contact is greatly increased; on the other hand, the biofilm provides a stable environment for the connection of pili, which is more conducive to conjugation [22,48]. It is unknown whether the conjugative transfer between the bacteria in the biofilm in vivo is more active than in vitro. Therefore, further research should pay attention to the effect of biofilm formation in vivo on conjugation and evaluate the risk of drug resistance transmission.

#### 2.2.3. Others

It was reported that physical factors such as mucins may also affect the spread of ARGs in the intestinal tract. Mucins in the intestine can provide adhesion sites for bacteria, which is conducive to the colonization of certain bacteria and further affects the composition of intestinal flora and the number of bacteria [49]. Therefore, mucin, as a physical factor widely distributed in the intestine, may also affect the spread of ARGs. 

The rumen is a unique digestive organ of ruminants and an important location for the transfer of ARGs. Studies have shown that rumen protozoa can promote plasmid transfer from *Klebsiella pneumoniae* to *Salmonella* in vitro and in vivo [50]. This is attributed to the high conjugative density of donor and recipient bacteria in vacuoles after phagocytosis by rumen protozoa and the stress conditions caused by rumen protozoa. However, whether bacteria can survive in protozoa for a long time to support the occurrence of conjugation has not been clearly observed.

### 2.3. Chemical Factor

Exogenous compounds that affect the CF in vitro studies have been extensively reported. The question remains as to whether these compounds play the same role in vivo? Aside from exogenous compounds, there may be more complex chemical factors in vivo that affect the efficiency of HGT, such as host autacoid, bacterial metabolites and exogenous compounds (Figure 2, Table 1).

#### 2.3.1. Host Autacoid

Bile and gastric acid secretion play a role in gastrointestinal digestion. Studies have shown that colistin resistance hinders plasmid conjugation related to colistin resistance in vitro. However, this phenomenon was reversed in the digestive tract of mice, as bile salts and anaerobic conditions in the intestine had a positive effect on the conjugation of the *mcr-*1 plasmid [33]. In addition, bile is an amphiphilic detergent molecule [51], which can cause damage to the bacterial cell membrane and increase the permeability of the bacterial cell membrane, which is conducive to conjugative transfer. The secretion of gastric acid provides an acidic environment in the gastrointestinal tract. Some acid-resistant bacteria can conjugatively transfer at a lower frequency under this extremely acidic condition (pH 1.0–4.0) [52]. It was reported that either too high or too low a pH inhibits the conjugative transfer process in laboratory conditions or a simulated natural environment [45]. However, the pH in the simulated conjugative system is impossible to attain in vivo. Whether slight changes in pH in the gastrointestinal tract due to disease status or inflammatory response cause significant changes in the CF of the plasmid needs further investigation.

Hormones such as norepinephrine (NE) and melatonin regulate the physiological functions of the host. Norepinephrine is a catecholamine derived from tyrosine. Sympathetic synapses using NE as a neurotransmitter are distributed throughout the body. The physiological concentration of NE in the gastrointestinal tract has been found to reach 50 μM [53]. Interestingly, conjugation experiments have shown that 5 μM of NE significantly reduces the CF of the plasmid [54]. Melatonin, one of the hormones secreted by the pineal gland in the brain, helps to treat sleep disorders and schizophrenia and has anti-cancer effects [55,56,57]. It can inhibit conjugation within and across genera under various conditions [58]. In addition, a mouse model of intraperitoneal infection was constructed, further indicating that the inhibitory effect of melatonin on conjugation in vivo is still effective [58].

#### 2.3.2. Bacterial Metabolites

In vivo, some bacterial products can also affect conjugative transfer. Studies have shown that the *Enterococcus faecalis* sex pheromone cCF10 is necessary for the effective transfer of pCF10 plasmid in vivo [59]. In germ-free mice, the pheromone produced by the recipient bacteria increases the CF of plasmid [59]. Recent studies have shown that the metabolites of lactic acid bacteria reduce the growth rate of *E. coli* [60]. The mathematical model was used to simulate the conditions in the gastrointestinal tract of chickens. It was found that the metabolites of lactic acid bacteria led to the loss of conjugative plasmids, which limited the spread of ARGs in the intestinal tract of chickens [60]. Indole is usually used as a biochemical indicator of bacteria and is produced via the hydrolysis of tryptophan [61]. Studies have shown that 250 μm indole can significantly inhibit the intergeneric conjugation of pUCP24T plasmids, and 50 μm indole can reduce the intrageneric CF by >3-fold [15]. There are many bacterial species in the gastrointestinal tract, and the effects of different bacterial species on colonization and the effects of different bacterial metabolites on conjugation are worthy of further study.

#### 2.3.3. Exogenous Compounds

The exogenous compounds that are accessible to humans or animals may also play a role in conjugation in vivo. Studies have shown that the intake of artificial sweeteners in the diet increases the resistance of intestinal microorganisms to antibiotics and promotes the conjugation of drug-resistant plasmids (Table 1) [32]. Common preservatives, such as sodium nitrite, sodium benzoate and triclocarban added to food, medicine and personal care products promote CF, leading to the development and spread of antibiotic resistance in the environmental and gastrointestinal microbiota [20]. These preservatives significantly increase CF at sub-inhibitory concentrations but exert inhibitory effects on conjugation at inhibitory concentrations, and their concentration in the gastrointestinal tract and effects on conjugation need to be further confirmed. In addition, when unsaturated fatty acid 2-hexadecynoic acid (2-HAD) was added to the food of mice, it could effectively inhibit the conjugative transfer of plasmids in the intestine [62]. In addition, the non-antibiotic drugs ibuprofen, naproxen and propranolol promote the conjugation of RP4 plasmids at the community level in vitro [63]. Similarly, acetaminophen, a widely used antipyretic in daily life, can promote the intrageneric conjugative transfer of plasmids at low concentrations, including environmentally relevant concentrations. When the concentration of acetaminophen was 50 μg/mL, the CF of RP4–7 plasmid increased by approximately 2.5 times, and that of plasmids with animal-derived *mcr-*1 and *tet*(X4) increased by 1.25–2.5 times [64]. It is important to note that the effect of these oral agents on plasmid conjugation in the gastrointestinal bacterial community remains to be investigated.

Though a number of reports about conjugation in vivo have been published, the compounds that affect this conjugation still need to be revealed. However, the establishment of an in vivo conjugation model is limited by many factors. To accelerate in vivo research, there is an urgent need for an in vivo model that is widely recognized and easily generalized.

**Table 1:** Factors influencing conjugation in vivo.

Factors	In Vivo Model	Strain	Plasmid	Effects	Reference
Factors affecting the density of donor and recipient bacteria:					
Ampicillin	Murine gut	*E. coli* O80:H26 to *S.* Heidelberg	IncI2	Promote	[39]
Enrofloxacin	Chicken gut	*E. coli* E2 to *Salmonella* SE211	IncY	Promote	[34]
Penicillin	Rat gut	*K. pneumoniae* HN-1 to gastrointestinal tract *E. coli*	*bla*NDM-1-bearing plasmid	Promote	[38]
Physical factor:					
Temperature	Chicken meat	*mcr-1*-positive donors to *mcr-1*-negative recipients (designated as R7 and *E. coli* C600)	*mcr-1*-positive plasmid	37 °C > 25 °C	[43]
Biofilm	Simulation in vitro	/	/	Promotion	[22]
Rumen protozoa	Rumen protozoa	*K. pneumoniae* TCR2003 to *S. Typhimurium* SL1344	pGFP	Promotion	[50]
Mucin	Simulation in vitro	*K. pneumoniae* A2312NM to *K. pneumoniae* MP13	pHNSHP45	Inhibition	[33]
Chemical factor:					
pH	Simulation in vitro	*E. hormaechei* to *E. coli* BL21(DE3)	pET28a-EGFP	/	[52]
Bile salts	Simulation in vitro	*K. pneumoniae* A2312NM to *K. pneumoniae* MP13	pHNSHP45	Promotion	[33]
Norepinephrine (NE)	Gut (simulation in vitro)	*S. Typhimurium* 5678 to *E. coli* C600N		Promotion	[54]
cCF10	Murine gut	/	pCF10	Promotion	[59]
Metabolites of lactic acid bacteria	Chicken gut (simulation in vitro)	*E. coli* MG1655 to *E. coli* MG1655	IncI1	Inhibition	[60]
Indole	Simulation in vitro	*E. coli* SM10λπ-PAO1 and SM10λπ-EC600	pUCP24T	Inhibition	[15]
2-hexadecynoic acid (2-HDA)	Murine gut	*E. coli* MDS42 to *E. coli* MDS52	pOX-38	Inhibition	[62]
Melatonin	Mouse infection model	*E. coli* DH5α to *E. coli* EC600	RP4–7	Inhibition	[58]
Artificial sweeteners	Murine gut (simulation in vitro)	*E. coli* K-12 MG1655 to mice feces	pKJK5 plasmid	Promotion	[32]

## 3. Potential Mechanisms Influencing CF In Vivo

The density of donor and recipient bacteria, both intergeneric or intrageneric, is a key variable in the CF in vivo, especially in the gastrointestinal tract [36]. Therefore, any factor that causes disturbance in the conjugation environment may lead to changes in the density of the donor or recipient bacteria, thereby affecting the conjugation transfer. In addition, the factors affecting conjugative transfer will also act on the conjugative process and directly affect CF.

### 3.1. Bacterial Colonization

The colonization of plasmid-carrying pathogens in vivo, especially in the gastrointestinal tract, is the fundamental factor affecting the density of donor and recipient bacteria. The main mechanism is related to the colonization resistance (CR) of intestinal symbiotic flora and the invasion of pathogenic bacteria. In addition, intestinal inflammation can also alter bacterial colonization. 

The gut has an extremely dense and complex microbial community, which maintains gut homeostasis. Some intestinal bacteria can resist the colonization and invasion of pathogens, which is known as colonization resistance [65,66]. Conjugation relies on cell density, as it requires the direct contact of donor and recipient bacteria through the sex pilus to establish a connection bridge [67,68]. However, CR mediated by intestinal symbiotic microbiota decreases the colonization and density of intestinal pathogenic bacteria (donor cells), thereby inhibiting the plasmid-mediated conjugative transfer of ARGs [65,66]. 

Exogenous pathogens with strong colonization and invasion ability are more easily able to break through the CR and colonize in the intestine, and even invade into tissues. *Salmonella typhimurium* strains can colonize the intestinal lumen and survive for a long period in host tissues, which depends on the type-3 secretion system-2 (TTSS-2) encoded by Salmonella pathogenicity islands (SPIs) [67,69,70,71]. A recent study showed that invasive *S. typhimurium* can form tissue-persister reservoirs in host tissues, which are difficult to be removed by antibiotics [72]. In addition, *S. typhimurium* can re-seed the gut lumen from these reservoirs, which may transfer plasmids to other bacteria in the gut via conjugation or accept plasmids as the recipient, and subsequently, re-enter the tissue reservoirs via re-invasion [72]. This phenomenon may lead to the accumulation of antibiotic-resistant plasmids in host tissue reservoirs and eventually promote the spread of ARGs in the gut through the cycle of re-seeding, conjugation and invasion [67,72].

The inflammatory responses produced by the intestinal immune system are often observed in the gut, which may reduce the colonization density of the long-term colonized symbiotic microbiota and then reduce CRs to pathogens (Figure 2b) [29,73]. Thus, pathogenic bacteria acquire more ecological niches, and their colonization density significantly increases, making conjugation more likely occur.

Overall, healthy and stable intestinal symbiotic microbiota is conducive to producing better CRs, thereby inhibiting the colonization of pathogens, significantly reducing their population density. In addition, it is necessary to explore the molecular mechanisms that affect in vivo conjugation and fundamentally control the transmission of resistance genes in vivo.

### 3.2. The Process of Conjugation

**Figure 3:**
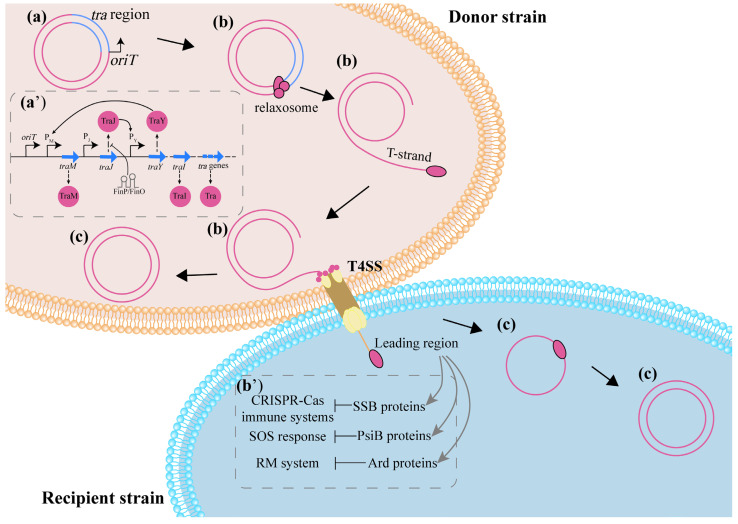
The process and regulation of conjugation. (**a**) When conjugation begins, the related genes on the conjugative plasmid begin to express and assemble the protein structures required for the conjugative process. (**a’**) Meanwhile, the expression of these genes is interrelated and follows cascade regulation. (**b**) Next, the relaxosome specifically recognizes and binds to the *oriT* site, and the double-stranded DNA (dsDNA) is unwound and becomes single-stranded DNA (ssDNA), which is recruited by the TraI relaxase protein, and enters the recipient bacteria through the type IV secretory system (T4SS) under the action of the type IV coupling proteins (T4CPs) located on the cell membrane. (**b’**) The leading region of the ssDNA that enters the receptor bacteria first encodes the corresponding protein to antagonize the host receptor defense response. (**c**) Finally, the ssDNA in the recipient bacteria is circularized under the action of the relaxase protein and re-forms into dsDNA by Rolling Circle Replication (RCR). Meanwhile, the single-stranded circular plasmid DNA in the donor bacteria also forms dsDNA in the form of RCR.

**Figure 4:**
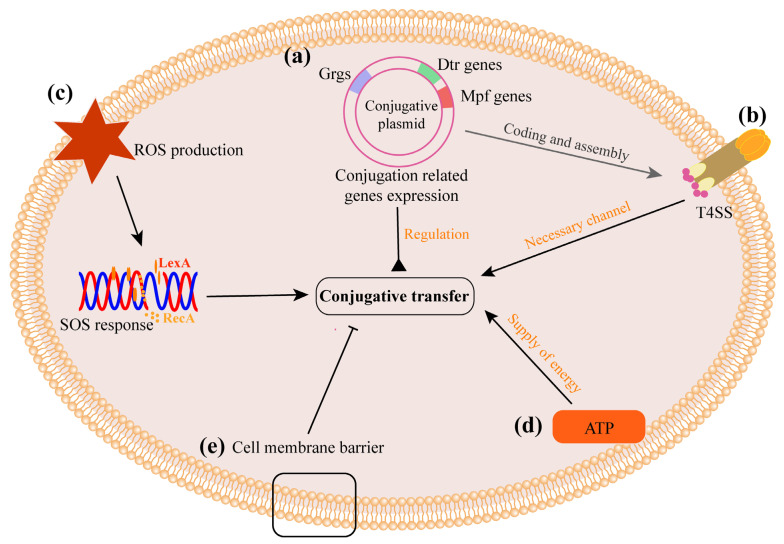
The potential mechanism affecting conjugation based on the basic process of conjugation. (**a**) The expression of conjugation genes on the plasmid regulates the conjugative process. (**b**) T4SS is a necessary channel for plasmid transfer and may be a potential target for conjugation. (**c**) An overproduction of ROS in bacterial cells causes DNA damage, which activates the SOS response and further affects the expression of conjugation genes, thereby promoting plasmid conjugation. (**d**) Adenosine triphosphate (ATP) synthesis provides energy for conjugation, which is essential for the conjugation process. (**e**) The cell membrane is the barrier of plasmid conjugation; the reduction in cell membrane permeability would inhibit conjugation.

The potential mechanism affecting conjugation is based on the basic process of conjugation. The key steps and regulatory mechanisms of F plasmid conjugation have been well reviewed [74,75]. However, the structure of the plasmid control circuit varies by plasmid type. Generally, in the donor bacteria, the expression of conjugation-related genes carried by the plasmid mobilizes the transfer of the plasmid, and the expression of *tra* gene follows a specific regulatory cascade [74]. In recipient bacteria, the expression of leading genes regulates plasmid colonization, including resistance to host bacterial defense clearance, DNA cyclization and rolling circle replication (Figure 3) [75]. Thus, any factors impacting the expression of conjugation-related genes would exert an effect on CF. In addition, bacterial reactive oxide species (ROS) production, SOS response, energy supply and cell membrane permeability can affect the process of conjugation and change the CF (Figure 4). 

#### 3.2.1. Interference in the Expression of Conjugation-Related Genes Directly Affects CF

Recently, most of the factors that affect conjugation have been studied using IncP plasmids [10,44,64]. Therefore, here, the IncP plasmid is used as a model to discuss how interference with the expression of these specific functional genes can directly affect conjugation.

Global regulatory genes (Grgs), DNA transfer and replication (Dtr) genes, and mating pair formation (Mpf)-related genes play a critical role in the plasmid-mediated conjugative transfer of ARGs [76,77]. The Grgs *korA*, *korB* and *trbA* negatively regulate conjugation and inhibit the expression of downstream genes [45]. Previous studies have shown that the combination of *korB* and *trbA* can significantly inhibit *trbBp* [78]. In addition, indole can promote the expression of *korA* and *korB*, further inhibiting the expression of *trbB* and *trfA* and eventually reducing the CF of pUCP24T plasmids [15].

The Dtr genes *trfA* and *traJ* play an important role in relaxosome formation during conjugation, thereby facilitating conjugation [79]. Studies have shown that four common non-nutritive sweeteners can promote the expression of the conjugation regulatory gene *traG* and the Dtr genes *traC1* and *traC2*, and the expression of *traG* contributes to the formation of the relaxosome–Mpf complex [80]. 

The Mpf system is a bridge between donor and recipient bacteria and an important channel for plasmid transfer [81]. The *trbB* gene is the primary promoter in the Mpf system. Melatonin can repress the expression of *trbB*, thereby reducing the CF of RP4 plasmids [58]. In addition, some pilus formation-related genes contribute to the formation of the Mpf system. For example, the regulatory genes *ecpA*, *fimH* and *yagI* related to pilus formation in donor bacteria contribute to conjugation [10,80]. Other genes encoded by plasmids (including *traA*, *traB*, *traF*, *traH*, *traL* and *traP*) can also contribute to the formation of the Mpf system for intercellular DNA transfer during bacterial conjugation [58,77,81,82]. 

T4SS is a macromolecular complex involved in substrate transport and pilus biogenesis and mediates conjugation in Gram-negative and a few Gram-positive bacteria, and it has been very well reviewed [83]. The proteins that constitute T4SS can be divided into three categories according to their corresponding functions: pilus proteins, translocation channel proteins and ATPases responsible for providing energy for pilus biogenesis and substrate transport [84]. Therefore, T4SS is a very important potential target for controlling conjugation and may be an effective strategy for preventing and controlling the HGT of ARGs.

In addition to the important conjugation-related genes, some other genes also play an indispensable role in regulating conjugation. A gene located in the Tra2 region of the InHI1 R27 plasmid (ORF R0009) encodes an immunoglobulin-like protein called RSP, which interacts with bacterial flagella and reduces bacterial cell motility, thus enabling conjugation [85,86]. In addition, a new transcriptional regulation gene, *pixR*, has been identified in the IncX4 pHNSHP23 plasmid. The expression product of *pixR* can directly regulate plasmid transfer by interacting with the promoter of the transfer operon, thereby increasing its transcriptional level and CF [87]. Therefore, the identification of such additional genes or proteins may offer important targets for controlling the plasmid-mediated spread of ARGs.

#### 3.2.2. ROS Production and SOS Responses Increase CF

ROS are the products of oxidative stress, which is a general term that encompasses oxygen free radicals (such as superoxide, peroxide and hydroxyl) and some non-radical oxidants (such as hydrogen peroxide [H_2_O_2_]) [88,89]. ROS are also generated during normal metabolic processes and participate in physiological and biochemical reactions in cells [89]. Excessive ROS production causes DNA damage in bacterial cells, thereby activating SOS responses, which can affect the expression of conjugation-related genes and promote conjugation. For example, exposure to silver ions and silver nanoparticles can cause an overproduction of ROS in bacteria, leading to cell membrane damage and DNA damage. As a result, the bacterial cell membrane barrier is disrupted to facilitate the conjugative transfer of plasmids [90]. Metal ions and nanomaterials generally alter CF by affecting the conjugation process between bacteria in natural environments such as the water environment. However, these compounds are easily enriched in the gastrointestinal tract and gills of aquatic animals. For example, whether the concentration of silver ions and silver nanoparticles will still affect the production of ROS in bacterial cells when they are enriched in vivo and the environment in vivo, and thus, affect the transfer of plasmids in vivo, remains to be further studied.

Studies have shown that oxidative stress plays an important role in the development of antibiotic resistance and the conjugative transfer of resistance plasmids [91,92]. DNA damage caused by an excessive production of ROS can guide SOS responses, thereby initiating DNA damage repair [93,94]. SOS responses are mainly regulated by two proteins, LexA (a transcriptional repressor) and RecA (a recombinase) [95]. In a steady state, LexA expression can inhibit SOS responses in bacterial cells. In the presence of DNA damage, RecA is activated to induce SOS responses [96]. In the presence of the integrating conjugative elements (ICEs) of the SXT family, SOS responses inactivate SetR, thereby relieving the inhibition of *setC* and *setD* expression and eventually increasing the CF of SXT [97]. In vivo studies have shown that artificial sweeteners, such as aspartame, saccharin, sucralose and acesulfame potassium, can cause an excessive production of ROS in bacterial cells and further promote the conjugative transfer of plasmids in the intestine [32].

In addition, conjugation can in turn induce SOS responses because SOS response-regulated DNA damage repair is triggered when ssDNA enters recipient cells during conjugation [98]. Therefore, the production of ROS and SOS responses in bacterial cells can promote the conjugation of plasmids to a certain extent, and the factors affecting the production of ROS and SOS responses in vivo, especially in the gastrointestinal tract, should be paid attention.

#### 3.2.3. Energy Supply Is Necessary for Conjugation

Adenosine triphosphate (ATP) plays an important role in conjugation by providing energy for the assembly of conjugation-related components, plasmid replication and transportation [99]. Intracellular ATP is mainly produced via aerobic respiration, such as via the tricarboxylic acid (TCA) cycle, electron transport chain and oxidative phosphorylation. 

Nicotinamide adenine dinucleotide (NADH) is an important electron donor in the bacterium’s electron transport chain [100]. Under the action of dehydrogenase and oxidase in the electron transport chain, protons are pumped out of the cell membrane, forming the proton motive force (PMF). Finally, protons enter the cell via ATP synthase channels, and the inorganic phosphate group combines with adenosine diphosphate (ADP) to form ATP [101]. Therefore, PMF is the main driving factor of ATP synthesis [102]. Studies have shown that melatonin can significantly inhibit the conjugative transfer of plasmids, and in the mouse infection model [58]. The mechanism study found that PMF was destroyed after exposure to melatonin, and ATP synthesis was inhibited in the recipient bacteria [58]. 

#### 3.2.4. Cell Membrane Is a Barrier to Plasmid Transfer

The bacterial cell membrane is semipermeable and composed of a phospholipid bilayer and membrane proteins. It plays an important role in nutrient absorption, the excretion of metabolic waste and protein, and material transport [103]. The cell membrane is a barrier that protects the intracellular environment from the extracellular environment and is an obstacle to the spread of ARGs between bacteria [104]. Increased permeability and disruption of the integrity of the bacterial cell membrane may provide more opportunities for the spread of ARGs. Damage to the bacterial cell membrane or an increase in permeability may be more conducive to the transfer of plasmids carrying ARGs between bacteria and increases CF. In addition, an overproduction of ROS may also increase permeability and cause damage to the bacterial cell membrane [93,105].

The underlying mechanisms change in the bacterial cell membrane may be related to the expression of membrane-related genes. For example, *ecnB*, *exbB* and *exbD* can regulate cell membrane stability and activate outer membrane transport [106], and the upregulation of some genes encoding major outer membrane proteins, such as *ompA*, *ompC* and *ompF*, can increase CF [19,107]. The outer membrane pore protein encoded by *ompF* plays an important role in cell membrane permeability. When *ompF* expression is inhibited, the pore protein channel is narrowed and blocked, leading to the obstruction of material transport and a significant reduction in CF [108,109]. Altogether, the integrity and permeability of the bacterial cell membrane and the expression of genes encoding membrane proteins play an important role in the plasmid-mediated conjugative transfer of ARGs. 

## 4. Conclusions and Outlook

In vivo, especially in the gastrointestinal tract, high bacterial density, suitable temperature and nutritional conditions create a “melting pot” of plasmid conjugation for the spread of antibiotic resistance. However, the diversity of bacterial species, the dynamics of bacteria in different spaces and the diversity of drug-resistant plasmids pose great challenges to studying the conjugation-mediated ARGs transmission in vivo. Thus, few studies have systematically investigated the factors and mechanisms that influence conjugation in vivo. 

Understanding the factors affecting the conjugation of drug-resistant plasmids in vivo and the dynamic process of the conjugation of donor and recipient bacteria in vivo is critical to reduce the spread and prevalence of ARGs. Thus, we call for more attention to be paid to in vivo conjugation, including the establishment of scientific and reliable in vivo research methods, the early warning of factors contributing to conjugation, the development of conjugation inhibitors, and in-depth exploration of the in vivo mechanisms affecting conjugation.

## Data Availability

No new data were created or analyzed in this study. Data sharing is not applicable to this article.
